# Being Small for Gestational Age: Does it Matter for the Neurodevelopment of Premature Infants? A Cohort Study

**DOI:** 10.1371/journal.pone.0125769

**Published:** 2015-05-12

**Authors:** Myriam Bickle Graz, Jean-François Tolsa, Céline Julie Fischer Fumeaux

**Affiliations:** Clinic of Neonatology and Developmental Unit, Department of Pediatrics and Pediatric Surgery, University Hospital and University of Lausanne, Lausanne, Switzerland; Hôpital Robert Debré, FRANCE

## Abstract

**Background:**

Whether being small for gestational age (SGA) increases the risk of adverse neurodevelopmental outcome in premature infants remains controversial.

**Objective:**

to study the impact of SGA (birthweight < percentile 10) on cognition, behavior, neurodevelopmental impairment and use of therapy at 5 years old.

**Methods:**

This population-based prospective cohort included infants born before 32 weeks of gestation. Cognition was evaluated with the K-ABC, and behavior with the Strengths and Difficulties Questionnaire (SDQ). Primary outcomes were cognitive and behavioral scores, as well as neurodevelopmental impairment (cognitive score < 2SD, hearing loss, blindness, or cerebral palsy). The need of therapy, an indirect indicator of neurodevelopmental impairment, was a secondary outcome. Linear and logistic regression models were used to analyze the association of SGA with neurodevelopment.

**Results:**

342/515 (76%) premature infants were assessed. SGA was significantly associated with hyperactivity scores of the SDQ (coefficient 0.81, p < 0.04), but not with cognitive scores, neurodevelopmental impairment or the need of therapy. Gestational age, socio-economic status, and major brain lesions were associated with cognitive outcome in the univariate and multivariate model, whereas asphyxia, sepsis and bronchopulmonary dysplasia were associated in the univariate model only. Severe impairment was associated with fetal tobacco exposition, asphyxia, gestational age and major brain lesions. Different neonatal factors were associated with the use of single or multiple therapies: children with one therapy were more likely to have suffered birth asphyxia or necrotizing enterocolitis, whereas the need for several therapies was predicted by major brain lesions.

**Discussion:**

In this large cohort of premature infants, assessed at 5 years old with a complete panel of tests, SGA was associated with hyperactive behavior, but not with cognition, neurodevelopmental impairment or use of therapy. Birthweight <10th percentile alone does not appear to be an independent risk factor of neurodevelopmental adverse outcome in preterm children.

## Introduction

Each year 15 million babies, or 1/10 babies, are born premature, of whom 1 million die and many suffer from lifelong disabilities[1]. Very preterm children, born before the 32^nd^ week of gestation, are especially at risk of long-term morbidities, due to numerous antenatal or neonatal variables. Among them, infants born with a birthweight below the 10^th^ percentile, or small for gestational age (SGA), are estimated to encompass 15 to 30% of very preterm infants[2]. These SGA infants are small either due to constitutional reasons, or have suffered from intrauterine growth restriction (IUGR), secondary to maternal, placental, fetal or environmental factors[3]. The etiology of SGA is frequently unknown in population studies and depends on antenatal information about intrauterine growth of the fetus and materno-fetal circulation. Many studies use the term intrauterine growth restriction (IUGR) indifferently for SGA and IUGR.

SGA and IUGR infants have been shown to be at risk of increased neonatal mortality and short- and long-term morbidities, in term or preterm born infants [4–7]. Imaging studies have shown alterations in brain structure and reduced brain volumes in IUGR preterm infants compared to appropriate birth weight controls [8, 9]. However, there are conflicting results as to long term neurodevelopmental outcome, with some studies showing no difference [10–12], whereas others reported increased levels of cognitive and behavioral difficulties [13, 14]. There is yet no clear explanation for this possible altered neurodevelopment.

Our aim was thus, in a cohort of children born before 32 weeks of gestation, to study the relative impact of SGA, defined as a birthweight below the 10^th^ percentile, on neurodevelopmental outcome at 5 years old, assessed with a panel of tests examining different aspects of child neurodevelopment. Our primary outcome was the neurodevelopmental status assessed by cognitive and behavioral scores and neurological examination. Our secondary outcome was an indirect evaluation of neurodevelopmental impairment, through the need of therapy.

## Methods

### Design

This study was nested in a population based longitudinal prospective cohort of premature infants hospitalized in a tertiary care neonatal intensive care unit.

### Population

**C**onsisted of all preterm infants admitted to the Clinic of Neonatology of the University Hospital in Lausanne, Switzerland between 01.01.2000 and 31.12.2005. Patients with severe brain malformations, lethal malformations, or genetic disorders known to interfere with neurodevelopment were excluded. As is the case in all tertiary care centers taking part in the Swiss Neonatal and Follow-up group, specialized neurodevelopmental follow-up was offered to all families when the infants left the Clinic, and the families were informed of the aims of this follow-up, which was to offer early detection and treatment of developmental issues.

### Ethical statement

According to the Swiss law during the study period, no written informed consent was necessary for retrospective observational studies. The local Human Research Ethics Committee (Commission cantonale d’éthique de la recherche sur l’être humain) and the hospital direction granted a general approval for research on coded or anonymized retrospective data. Families were orally informed of the possibility of research using the data, and children were included in the database if the parents did not refuse it. Data were collected in a specific database accessible only to clinicians involved in the care of the patients. To analyze the dataset, an extraction was made, the result of which was coded data.

### Data collection

All perinatal and follow-up data were collected in a specific database: Perinatal data known from the literature to be associated with developmental outcome of premature infants were retrieved from the child’s file and entered in the database at the moment of the first follow-up visit. Prenatal data were self-reported mother’s smoking during pregnancy, gender, multiple pregnancy and parental socioeconomic status according to Largo[15], which entails a 6 point scale for each parent, with recorded mother’s education (1 = university and 6 = special or no schooling) and father’s occupation (1 = leading position and 6 = unskilled labor). The scores are thus distributed from 2–12, and were categorized as high (2–5), middle (6–8) and low (9–12) socioeconomic status.

The postnatal data collected were: gestational age in completed weeks of gestation, assessed with best obstetric estimate comprised of the mother’s last menstrual period when available, and with early first trimester ultrasound scan, birthweight, and birthweight percentile. SGA was defined as being <10th percentile for the weight based on the growth curves by Voigt et al[16]. Birth asphyxia according to Apgar score (Apgar less than 6 at 5 minutes) and to umbilical cord blood pH (arterial cord blood pH less than 7.0), presence of proven sepsis defined as clinical signs and at least one positive blood culture at any point during the hospital stay, proven necrotizing enterocolitis (NEC), bronchopulmonary dysplasia defined as a need for supplemental oxygen or ventilatory support at 36 weeks postmenstrual age (BPD), major brain lesions (intraventricular hemorrhage grade III or more, cystic periventricular leucomalacia according to Papile)[17]

Follow-up data were entered in the database at the subsequent visits, at the ages of 6 and 18 months (age since term), and at 3.5 and 5 years old, the data about the latter examination were used for this study; they included cognitive and behavioral score, as well as neurological assessment and record of the use of therapy, as described below.

### Primary outcomes

#### Cognitive outcome at 5 years old

Cognitive development was assessed with the French version of Kaufman Assessment Battery for Children (K-ABC)[18], which entails 3 subscales of sequential processes, simultaneous processes, and composite mental processes (CMP), judged to be an equivalent of an intelligence quotient. This last score has an expected mean of 100 and a standard deviation of 15. For children known to have major developmental problems, the psychologist may have chosen to use another standardized test, such as Wechsler Intelligence for preschool and primary school, third edition (WPPSI-III)[19], also with a mean at 100 and a standard deviation of 15, or rarely McCarthy Scales of Children’s Abilities[20, 21] of which the mean is 100 and the standard deviation 16. Because of the different standard deviations, the results of the 3 tests were converted to z-scores, which were used for the analysis, as has been previously done and published [22].

#### Behavioral outcome

Caregivers filled in the French version of the “Strengths and Difficulties Questionnaire”(SDQ), a validated tool to assess different aspects of behavior[23]. This questionnaire consists of 25 questions, and the results entail five subscales of emotional, behavior, hyperactivity, relational, and prosocial issues, and a total problem score [24].

#### Neurological outcome

Children were subjected to a detailed neurological examination to assess neuromotor function and exclude cerebral palsy, vision and hearing were tested. Cerebral palsy was defined as a disorder of movement and/or posture and of motor function, due to a non-progressive interference, lesion, or abnormality of the developing/immature brain [25]. Neurodevelopmental impairment consisted in a composite endpoint of IQ z-score < -2 (by definition mental retardation), or cerebral palsy any grade [26], or severe hearing (corrected or not by hearing aids) or vision problems (blindness in at least one eye), and normal outcome consisted in none of the above.

### Secondary outcome: Use of therapy at 5 years old

Information about use of medico-educational therapies at the moment of the 5 year old examination were collected, and were divided into no treatment, one treatment (for example speech and language therapy), or multiple treatments. These therapies were mainly prescribed by the professionals in the Unit.

### Statistical methods

Data were analyzed with Stata version 13 (Statacorp, Texas, USA). The population characteristics were reported in means (standard deviations) for continuous variables and frequencies (%) for binary and categorical variables. Differences among subgroups of infants with or without SGA were assessed using t-tests and chi-square tests, respectively.

The z-scores of the cognitive scores and the behavioral scores were analyzed with univariate linear regression models first. Risk factors that had a p-value below 0.2 in the univariate analysis were explored with multivariate linear regression models, using a step forward and a step backward methodology. Results are reported in the table as coefficients with the 95% confidence intervals, additionally beta weights were calculated to evaluate the size of the effect, but are not reported for all variables as they were weak.

Neurodevelopmental impairment was analyzed with simple univariate logistic regression, using a step forward and a step backward methodology, retaining for the multivariate analysis the variables that had a p value < 0.2.

Finally, the analysis of use of therapy implied a multinomial logistic regression, first in a univariate model, and then in a multivariate model. Again, we only included in the multivariate model the variables that had a p-value <0.2 in the univariate model. All the results of the logistic regressions are given as coefficients with 95% confidence intervals and p values.

## Results

**Fig 1:**
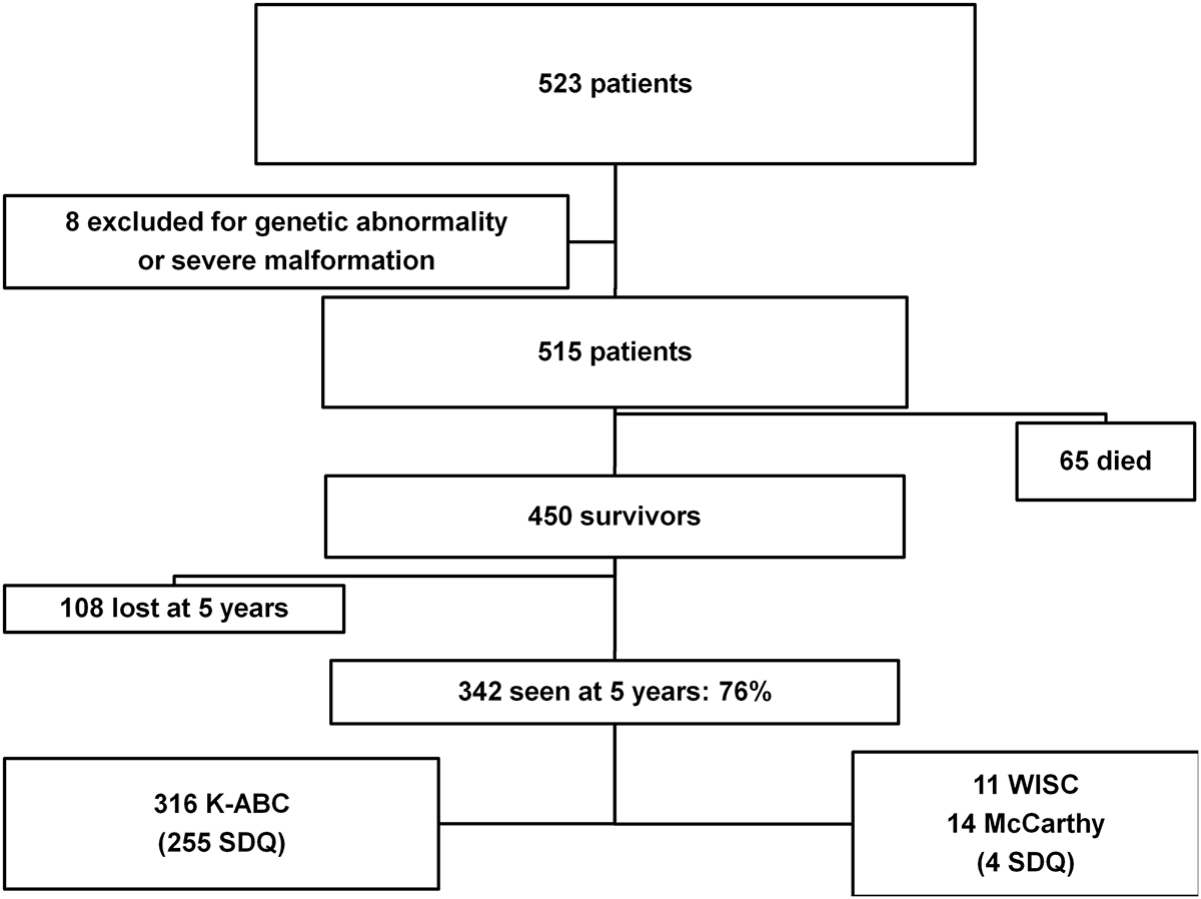
Flow chart of the study population.

During the six years of the study period, 523 patients born before 32 weeks of gestation were hospitalized in our tertiary care Neonatology Clinic, with a mortality rate of 12.4% (AGA 11.2% and SGA 14.7%, p = 0.351), as shown in Fig 1.

The rate of follow-up was 342/450 (76%) at 5 years old. There was no statistically significant difference in most of the neonatal pathologies between lost or followed infants except for multiple births (who tended to be less followed), and children who had suffered from BPD who were more often followed. The population had 54/342(15.8%) of SGA infants. The other main neonatal characteristics are described in Table 1.

**Table 1:** Population characteristics.

	Alln = 342	SGA^1^54 (15.8%)	AGA^2^288 (84.2%)	p value*AGA versus SGA
**Antenatal characteristics**				
Antenatal steroids (n, %)	218/333 (65)	37/54 (68)	181/279 (65)	0.606
Outborn (n, %)	61 (17.9)	8 (14.8)	53 (18.5)	0.521
Male gender (n, %)	175 (50.8)	28 (51.8)	146 (50.7)	0.876
Multiple gestation (n, %)	78 (22.3)	10 (18.5)	68 (23.6)	0.413
Gestational age (weeks) (mean, SD, range)	28.4(6.8,24–31.8)	28.5(6.8,24–31.8)	28.6(2,24–31.8)	0.640
**Neonatal characteristics**				
Birthweight (g)(mean, SD, range)	1158(348,380–2280)	784(191,380–1415)	1228(325,650–2280)	< 0.001
Asphyxia (Apgar < 6 at 5 min.) (n, %)	94 (28.1)	19 (35.2)	75 (26.8)	0.209
Asphyxie (pH<7.0) (n, %)	17 (5.5)	1 (1.9)	16 (6.3)	0.209
Proven sepsis (n, %)	48 (14.0)	8 (14.8)	40 (13.9)	0.857
Major brain lesions (n, %)	42 (15.8)	9 (22.0)	33 (14.6)	0.252
Proven enterocolitis (n, %)	14 (4.0)	8 (14.8)	6 (2.0)	< 0.001
Bronchopulmonary dysplasia (n, %)	102 (30.0)	26 (49.0)	76 (26.6)	0.001
**Maternal characteristics**				
Largo score (mean, SD, range)	6.6(2.5, 2–12)	5.9(5.8, 2–12)	6.8(6.8, 2–12)	0.014
Smoking during pregnancy (n, %)	67 (21.7)	16 (31.4)	51 (19.8)	0.066

*p values were calculated by t-tests for continuous variables and by the chi2 test for categorical or binary outcomes.

^1^: SGA: small for gestational age, birthweight< percentile 10.

^2^: AGA: appropriate for gestational age, birthweight> percentile 10

### Primary outcomes

#### Cognitive outcome analyzed with z-score

Patients were examined at a mean age of 65 months (SD 4.6, range 45–87). Cognitive score, assessed with the K-ABC, WPPSI-III or McCarthy tests showed a mean z-score of- 0.4, SD 1.2, with an approximately normal distribution, and no statistically significant difference between SGA (-0.56) and AGA (-0.38) (p = 0.316). There were 27/342 (7.9%) of children with a z-score <- 2, corresponding to the definition of mental retardation. Table 2 shows the results of the linear regression analysis of the cognitive z-scores. There was no statistically significant association between SGA and cognitive outcome. Univariate regression showed an association of gestational age (coefficient 0.02 per additional day with a p value < 0.001, and thus 0.14 per additional week of gestation, which would be 2 points of intellectual quotient per week), proven BPD, sepsis, major brain lesions and socio-economic status with cognitive outcome. In the multivariate model, the neonatal variables associated with cognitive development were gestational age, major brain lesions and socio-economic status. The global effect of this model was still moderate, with r2 at 0.2, and the size of the effects, as measured by beta weights, was weak to moderate (0.19 for gestational age, 0.26 for major brain lesions). Most of the variables had a negative effect on the cognitive score, except for additional gestational days.

**Table 2:** Regression analysis of cognitive outcome.

	Univariate		Multivariate	
	Coefficient (95% CI)	p value	Coefficient (95% CI)	p value
**Antenatal characteristics**				
SGA^1^	-0.18 (-0.54–0.17)	0.316	-0.22(-0.59 0.15)	0.239
**Gestational age**	**0.02 (0.01–0.03)**	**<0.001**	**0.01 (0.01 0.03)**	**0.003**
Multiple gestation	0.21 (-0.10–0.52)	0.187	-0.03 (-0.35 0.29)	0.851
Male gender	-0.05 (-0.31–0.21)	0.716	---	---
**Neonatal characteristics**				
Asphyxia (pH)	-0.19 (-0.79–0.42)	0.525	---	---
Asphyxia (Apgar)	-0.34 (-0.63–0.05)	0.022	-0.08 (-0.39 0.22)	0.603
Necrotizing Enterocolitis	-0.31(-0.97 0.34)	0.349	---	---
Sepsis	**- 0.5 (-0.88–0.13)**	**0.008**	-0.29 (- 0.68 0.09)	0.140
BPD^2^	**- 0.35 (-0.63–0.07)**	**0.015**	-0.08 (- 0.41 0.24)	0.606
**Major brain lesions**	**-1.02 (-1.41–0.64)**	**<0.001**	**- 0.91(-1.33–0.49)**	**<0.001**
**Characteristics related to the mother**				
**Socio-economic status (ref: high)**				
**Middle**	- 0.61 (-0.91–3.17)	**<0.001**	**-0.53 (- 0.85–0.22)**	**0.001**
**Low**	- 0.98 (-1.35–0.61)	**<0.001**	**- 0.99 (-1.39–0.59)**	**<0.001**
Smoking	- 0.23 (-0.57 0.10)	0.169	-0.10 (- 0.43 0.22)	0.532

^1^: SGA: Small for gestational age.

^2^: BPD: bronchopulmonary dysplasia.

Values are coefficients (95% confidence interval) calculated with linear regression. The variables with p< 0.2, were retained for the multivariate regression, except for SGA, retained in all the analysis.

#### Behavioral outcome

Behavior was assessed with the SDQ, available for 259 children, of which was extracted a total problem score and five subscales. SGA was associated with the hyperactivity subscale (coefficient 0.81, p < 0.04), but with no other scale, as shown in Table 3.

**Table 3:** Strengths and Difficulties results.

Scores (mean)	All	SGA	AGA	p value
**Total score**	9.38	10.07	9.25	0.346
**Emotional symptoms**	2.04	2.21	2.00	0.524
**Conduct problems**	1.92	2.16	1.87	0.280
**Hyperactivity-inattention**	3.63	4.30	3.5	0.033
**Peer problems**	1.48	1.43	1.49	0.819
**Prosocial behavior**	8.26	8.57	8.19	0.173

Univariate analysis showed a significant association between male gender (coefficient 0.71, p<0.02), BPD (coefficient 0.67, p<0.03), and asphyxia based on a cord blood pH below 7.0 (coefficient -1.49, p<0.03) with the hyperactivity subscale. In the multivariate analysis, only male gender and SGA were statistically significant (SGA, coef. 0.77, p = 0.042, male gender (coef. 0.69, p = 0.014). There was a statistically significant association between multiple pregnancy and the relational score (coef. -0.49, p = 0.033, better relational abilities in multiplets), and gender and the prosocial score (coef. - 0.47, p = 0.021, better prosocial abilities in girls). Univariate analysis did not show any statistical association between prenatal or postnatal factors and the total score, nor the emotional or the behavioral subscale.

#### Neurological outcome

Neurological examination revealed cerebral palsy in 20/345 (5.8%) of the examined children, deafness in 3/345 (0.87%), there were no blind children in this population. The risk of neurodevelopmental impairment, defined as cerebral palsy, mental retardation, blindness or deafness, was explored with logistic regression, using the same perinatal variables. Being SGA was not associated with neurodevelopmental impairment, as shown in Table 4. The variables gestational age, asphyxia, and major brain lesions, were significantly associated with neurodevelopmental impairment in both the univariate and the multivariate models, whereas low socio-economic status was only in the univariate model. In the multivariate model, the significant predictors of neurodevelopmental impairment were thus tobacco consumption during pregnancy, gestational age, major brain lesions and birth asphyxia, defined with a cord blood pH below 7.0.

**Table 4:** Neonatal predictors of neurodevelopmental impairment.

	Univariate		Multivariate	
	Coefficient (95% CI)	p value	Coefficient (95% CI)	p value
**Antenatal characteristics**				
SGA^1^	-0.12 (-1.03 0.79)	0.795	-0.39 (-1.64 0.86)	0.541
**Gestational age (days)**	**0.03 (-0.05 0.01)**	**0.008**	**-0.03 (-0.07–0.01)**	**0.047**
Multiple gestation	0.44 (-1.29 0.41)	0.308	---	---
Male gender	0.27 (-0.38 0.91)	0.420	---	---
**Neonatal characteristics**				
**Asphyxia (pH)**	**1.18 (0.08 2.28)**	**0.035**	**1.44 (0.01 2.88)**	**0.050**
Asphyxia (Apgar)	0.29 (-0.39 0.98)	0.402	---	---
Necrotizing Enterocolitis	0.71(-0.60 2.03)	0.289	---	---
Sepsis	0.55 (-0.25 1.36)	0.177	-0.49 (-1.62 0.63)	0.392
BPD^2^	0.52 (-0.14 1.19)	0.121	-.09 (-1.07 0.88)	0.845
**Major brain lesions**	**2.80 (2.04 3.56)**	**<0.001**	**3.07 (2.09 4.06)**	**< 0.001**
**Characteristics related to the mother**				
Socio-economic status (ref: high)				
Middle	0.38 (-0.47 1.23)	0.384	0.29 (-0.80 1.38)	0.600
Low	-0.95 (0.01 1.89)	0.047	1.15 (-0.12 2.43)	0.076
**Smoking**	**0.73 (-0.01 1.46)**	**0.051**	**1.12 (0.17 2.07)**	**0.020**

^1^: SGA: Small for gestational age.

^2^: BPD: bronchopulmonary dysplasia.

Values are coefficients (95% confidence interval) calculated with logistic regression. The variables with p< 0.2, were retained for the multivariate regression, except for SGA, retained in all the analysis.

### Secondary outcome: Need of therapy

Information about use of therapy at five years old was available for 331/342 (96.8%) followed children. Use of therapy was explored with multinomial logistic regression, as this item was broken down in 2 endpoints, use of one type of therapy (for example, physiotherapy, speech and language) or use of several therapies (for example physiotherapy and occupational therapy). Most of the children did not need any therapy (236/331, 71.3%), some needed one therapy at the age of 5 years old (57/331, 17.2%), and 38/331 (11.5%) needed several therapies.

As shown in Table 5, being SGA was not associated with increased use of using therapy, both in the univariate and in the multivariate model.

**Table 5:** Neonatal predictors of use of therapy.

SINGLE THERAPY					MULTIPLE THERAPIES			
	Univariate		Multivariate		Univariate		Multivariate	
	Coefficient (95% CI)	p value	Coefficient (95% CI)	p value	Coefficient (95% CI)	p value	Coefficient (95% CI)	p value
**Antenatal characteristics**								
**SGA** ^1^	0.49 (-0.24 1.23)	0.187	0.26 (-0.69 1.22)	0.586	0.14 (-0.80 1.08)	0.768	-0.12 (-1.39 1.15)	0.852
**Gestational age (days)**	-0.03 (-0.05–0.01)	0.002	-0.03 (-0.06–0.01)	0.026	-0.01(-0.04 0.01)	0.159	-0.02 (-0.05 0.01)	0.181
**Multiple gestation**	0.19 (-0.48 0.87)	0.580			0.41 (-0.35 1.18)	0.288		
**Male gender**	0.38 (-0.19 0.97)	0.195	0.28 (-0.42 0.98)	0.435	0.38 (-0.30 1.07)	0.277	0.72 (-0.15 1.59)	0.107
**Neonatal characteristics**								
**Asphyxia (pH)**	1.19 (0.08 2.29)	0.035	1.47 (-0.08 2.94)	0.049	0.86(-0.51 2.24)	0.220	1.53 (-0.46 3.53)	0.132
**Asphyxia (Apgar)**	0.46 (-0.16 1.08)	0.148	0.19 (-0.64 1.04)	0.647	-0.15 (-0.95 0.65)	0.712	-1.15 (-2.45 0.14)	0.080
**Necrotizing Enterocolitis**	1.86 (0.68 3.05)	0.002	1.71 (0.20 3.21)	0.026	0.22 (-1.95 2.39)	0.841	0.17 (-2.61 2.27)	0.891
**Sepsis**	0.11 (-0.72 0.95)	0.790			0.43 (-0.46 1.34)	0.342		
**BPD** ^2^	0.90 (0.30 1.50)	0.003	0.31 (-0.48 1.10)	0.445	0.65 (-0.06 1.36)	0.073	0.32 (-0.67 1.31)	0.527
**Major brain lesions**	0.98 (0.18 1.79)	0.016	0.26 (-0.81 1.33)	0.635	1.73 (0.92 2.55)	<0.001	2.06 (1.03 3.09)	< 0.001
**Characteristics related to the mother**								
**Socio-economic status (ref: high)**								
**Middle**	-0.01 (-0.66 0.64)	0.981	0.41 (-0.42 1.25)	0.334	-0.70 (-1.58 0.18)	0.122	-0.99 (-2.04 0.05)	0.062
**Low**	-0.62 (-1.62 0.38)	0.228	-0.84 (-2.31 0.62)	0.258	0.79 (-0.06 1.65)	0.070	0.79 (-0.27 1.87)	0.145
**Smoking**	0.04 (-0.69 0.79)	0.901	0.11 (-0.74 0.97)	0.791	0.54 (-0.24 1.32)	0.176	0.88 (-0.07 1.84)	0.072

Values are coefficients (95% confidence interval) calculated with multinomial logistic regression. The variables with p< 0.2 for single or multiple therapies were retained for multivariate regression.

^1^: SGA: small for gestational age, birthweight < percentile 10.

^2^: BPD: bronchopulmonary dysplasia.

The neonatal variables associated with the use of a single therapy were different from those of multiple therapies. The multivariate multinomial model was highly significant (p< 0.001), and showed that gestational age, necrotizing enterocolitis and birth asphyxia were associated with the use of a single therapy, whereas the need for multiple therapies was solely predicted by major brain lesions.

## Discussion

In this large cohort of very premature infants, our aim was to evaluate the relative impact of being born SGA on a list of outcomes describing different aspects of child development. SGA was associated with hyperactivity symptoms at the age of 5 years old, but was not associated with cognitive scores, nor with neurodevelopmental impairment or with the use of therapy.

Behavior was assessed with the Strengths and Difficulties Questionnaire, a validated tool exploring different aspects of it. None of the neonatal variables was associated with the total difficulty score. Being SGA did have an impact on behavioral issues, with higher scores on the hyperactivity scale. This result is similar to the results of a recent publication comparing SGA versus AGA premature infants in a cohort study[13], as well as those of a large Finish study[27]. Male gender was a risk factor for hyperactive behavior as well, as is described in the general population[28]. A known risk factor for behavioral issues in term children[29], smoking in pregnancy, showed no association with the SDQ in this population. This is in accordance with the results of a recent study reporting an associations of birthweight with inhibitory control and brain volumes in term born adolescents, but no effect of maternal smoking[30].

Cognitive development in our population was mainly associated with gestational age and socio-economic status. The impact of social factors has been shown in other studies [19, 31] especially for premature born infants[32, 33]. There was an association of birth asphyxia in the univariate model, which disappeared in the multivariate model. We found no association of SGA with cognitive outcome, contrary to Kok et al[34], or McCarton et al, who published cohort studies in the nineties with similar definitions (birthweight< 10^th^ percentile)[35].

Neurodevelopmental impairment This third outcome, defined by the presence of cerebral palsy, mental delay, blindness or deafness was not associated with being SGA in our study contrary to the above-mentioned study by Morsing, et al. Gestational age and severe brain lesions were major determinants of neurodevelopmental impairment, such as is usually reported in the literature, as well as asphyxia, which is rarely mentioned in premature infants. A low socio-economic status was significantly associated with impairment in the univariate model, but not in the multivariate model. Fetal smoking exposition was associated with impairment solely in the multivariate model.

Therapy Finally, we chose to assess the need of therapy, that can be present in up to 36% of a typical term population when only one therapy is needed[36]. We distinguished patients who needed several therapies, who represent the more impaired children, from those needing only one therapy. The risk factors were clearly different in these two populations; gestational age, necrotizing enterocolitis and birth asphyxia increased the risk of need of one therapy, whereas only major brain lesions were associated with multiple therapies. Although the usefulness of many forms of therapy has not been clearly demonstrated yet, therapy is generally considered useful by clinicians. It can thus be a good indirect indicator of the special needs of a child, as well as of the additional burden for families and society.

Among other important risk factors and potential confounders, tobacco deserves special attention. A relationship between smoking and socio-economic status is often reported, and some authors postulate that the impact of socio-economic status on cognitive outcome could be partially mediated by tobacco[37]. In our study this relationship was not verified, and we found no association between tobacco and cognitive or behavioral outcome, but there is nevertheless a significant impact on neurodecvelopmental impairment. Unfortunately, the information about smoking did not include quantitative data, such as number of cigarettes per day, which could have led to different results. However, tobacco during pregnancy is certainly the main risk factor that could be actively prevented or limited in this population.

### Limitations

Although the data were prospectively collected in an ad hoc database, the retrospective nature of the analysis involves some risk of bias. The follow-up rate of 76% is another potential source of bias; the dropouts showed that they tended to come more from multiple births, and were slightly less ill in the neonatal period (less BPD). Another limitation is the use of different cognitive scores at the age of five years. Children tested with tests other than the K-ABC had more developmental issues, and there was a significant difference between the mean scores obtained with the K-ABC or the other tests. We therefore chose not to exclude these more impaired children from our analysis, and combined all the results with z-scores, as has been previously published[22].

It is also important to note that, in our study, the definition of SGA was a birth weight < 10^th^ percentile based on Voigt, et al curves[16], which is one of the commonest used in the literature, but is not the only one [38]. The use of the 10^th^ percentile alone does not allow to identify infants especially at risk for adverse neurodevelopmental outcome; however, it is possible that definitions or classifications that better discriminate SGA form IUGR would be more predictive. For example, the use of customized growth curves for neonates, which take into consideration mother’s weight, height, and parity, could be more discriminative, as could be the precise documentation of intrauterine fetal growth restriction (IUGR), and of Doppler information [39, 40]. The prospective collection of these variables should contribute to better understand the risk factors of premature infants.

In conclusion, SGA (defined as birthweight < 10^th^ percentile) in premature infants seems to have a slight impact on neurodevelopment at the age of 5 years old, with more hyperactivity symptoms. No association was found with cognitive development, severe neurodevelopmental impairment or the need of therapy. The impact of IUGR identified with more specific approaches remains to be elucidated. The most potent predictors of neurodevelopment were gestational age, major brain lesions, socioeconomic status, birth asphyxia, and tobacco exposition during pregnancy. A future target of research, aimed at evaluating what is changeable, could be educational intervention in low-income families of premature children, especially for SGA children, and of course smoking counseling for mothers-to-be.
